# Influence of fluid accumulation on major adverse kidney events in critically ill patients – an observational cohort study

**DOI:** 10.1186/s13613-024-01281-7

**Published:** 2024-04-08

**Authors:** Debora M. Hofer, Livio Ruzzante, Jan Waskowski, Anna S. Messmer, Carmen A. Pfortmueller

**Affiliations:** Department of Intensive Care Medicine, Inselspital, Bern University Hospital, Freiburgstrasse 18, Bern, CH-3010 Switzerland

**Keywords:** Fluid management, Fluid overload, Renal recovery, Persistent renal failure, MAKE30

## Abstract

**Background:**

Fluid accumulation (FA) is known to be associated with acute kidney injury (AKI) during intensive care unit (ICU) stay but data on mid-term renal outcome is scarce. The aim of this study was to investigate the association between FA at ICU day 3 and major adverse kidney events in the first 30 days after ICU admission (MAKE30).

**Methods:**

Retrospective, single-center cohort study including adult ICU patients with sufficient data to compute FA and MAKE30. We defined FA as a positive cumulative fluid balance greater than 5% of bodyweight. The association between FA and MAKE30, including its sub-components, as well as the serum creatinine trajectories during ICU stay were examined. In addition, we performed a sensitivity analysis for the stage of AKI and the presence of chronic kidney disease (CKD).

**Results:**

Out of 13,326 included patients, 1,100 (8.3%) met the FA definition. FA at ICU day 3 was significantly associated with MAKE30 (adjusted odds ratio [aOR] 1.96; 95% confidence interval [CI] 1.67–2.30; *p* < 0.001) and all sub-components: need for renal replacement therapy (aOR 3.83; 95%CI 3.02–4.84), persistent renal dysfunction (aOR 1.72; 95%CI 1.40–2.12), and 30-day mortality (aOR 1.70; 95%CI 1.38–2.09), p all < 0.001. The sensitivity analysis showed an association of FA with MAKE30 independent from a pre-existing CKD, but exclusively in patients with AKI stage 3. Furthermore, FA was independently associated with the creatinine trajectory over the whole observation period.

**Conclusions:**

Fluid accumulation is significantly associated with MAKE30 in critically ill patients. This association is independent from pre-existing CKD and strongest in patients with AKI stage 3.

**Supplementary Information:**

The online version contains supplementary material available at 10.1186/s13613-024-01281-7.

## Background

Intravenous fluid administration is generally the first line therapy in patients with signs of inadequate tissue perfusion and can be lifesaving [1, 2]. One goal of fluid administration in the critically ill is to improve renal perfusion and thereby prevent or limit acute kidney injury (AKI) [3, 4]. However, despite the potential beneficial effects, fluid therapy can also substantially harm critically ill patients [2]. Excessive fluid administration alone or in combination with reduced urinary output leads to accumulation of extracellular fluid and the formation of interstitial edema as capillary leakage is often present in the critically ill [2, 5, 6]. Fluid accumulation (FA) can worsen organ function due to several mechanisms including impairment of oxygen and metabolite diffusion, toxin, and waste product clearance as well as obstruction of venous outflow and lymphatic drainage [2].

FA is a common state in the critically ill with up to every second patient developing FA, depending on their underlying disease profile, during the first five days after intensive care unit (ICU) admission and is associated with multiple adverse effects [7, 8]. Among others, FA is associated with a worse renal function [9, 10]. Some investigations indicate a relationship between FA and increased incidence and severity of AKI during ICU stay [10–12]. AKI is associated with an increased hospital mortality as well as negative long-term effects such as an increased risk of death, cardiovascular events and development or progression of chronic kidney disease [13, 14]. Discussed mechanisms for the association between FA and AKI include increased renal interstitial pressure secondary to renal-venous congestion or intra-abdominal hypertension results in intrinsic renal compartment syndrome, and thus transiently reduced renal perfusion pressure and renal function [6, 8, 15, 16]. In addition, AKI itself contributes to FA due to reduced urinary output, producing a vicious cycle [3, 9, 12].

However, there are only few data on mid-term renal outcome, and the available data often apply to very specific subgroups of ICU patients (i.e., patients with AKI on renal replacement therapy (RRT)) [5, 7, 17].

Hence, the primary outcome of this study was to investigate the association between FA at ICU day 3 and major adverse kidney events in the first 30 days after ICU admission (MAKE30) in a mixed ICU patient population. As secondary outcomes, we aimed to analyze the impact of FA on the sub-components of MAKE30 as well as on trajectory of creatinine values in patients with/without FA.

## Methods

### Study setup and design

**Fig. 1:**
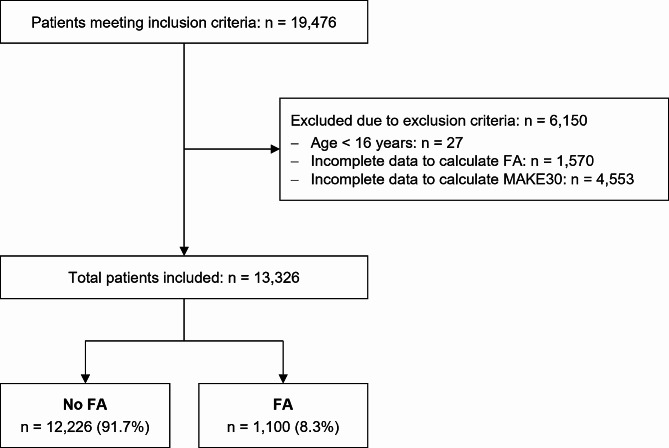
STROBE Flowchart. *FA*: fluid accumulation; *MAKE30*: major adverse kidney events in the first 30 days after intensive care unit (ICU) admission

The data for this single-center retrospective cohort study originates from the Inselspital, University Hospital of Bern, Switzerland. Data were collected from patients admitted to our clinic between January 2014 and June 2018. All patients admitted to the ICU or intermediate care unit (IMC) during the study period were eligible for study inclusion. We excluded patients under the age of 16 years or with incomplete data to calculate FA and/or MAKE30. The study adheres to the applicable STROBE guidelines, see Fig. 1 for the STROBE flowchart.

### Data collection

The data used for this analysis were extracted from the electronic medical databases of our hospital (SAP ERP 6.07/Inselspital Bern © SAP Schweiz 2018, Centricity Critical Care 8.1 © GE Electric Company 2018, Xserv.4 R19.3 © ixmid GmbH 2020, ipdos V7.16, © CompuGroup Medical Schweiz AG) and allocated to a large database on fluids, FA, and electrolyte disorders in critically ill patients. There are two other published projects out of this database [18, 19].

Among others, the database provides data on patient characteristics, medical history, and patient condition at ICU admission and during ICU stay. In addition, data on therapies (e.g., fluid input and output, RRT) as well as laboratory values and outcome data were collected. Furthermore, mortality data was extracted from the Swiss National Death Registry (Zentrales Sterberegister, ZAS).

### Objectives

The primary objective of this study was to investigate the association between FA at ICU day 3 and MAKE30.

Secondary, we assessed the relationship between FA and the individual components of MAKE30 (RRT, persistent renal dysfunction, mortality). In addition, we analyzed the trajectory of creatinine values in patients with/without FA over time and the development of FA in the first three days of ICU stay. The impact of FA on MAKE30 and its individual components in relationship to the stage of AKI and chronic kidney disease (CKD) as well as the impact of FA on creatinine trajectory over time were explored in the sensitivity analysis.

### Definitions

#### Fluid accumulation (FA)

To describe this pathological condition of overhydration, in line with recent publications, we used the term fluid accumulation (FA) [20, 21]. We used a weight-based approach and defined FA as a cumulative positive fluid balance (CFB) greater than 5% of bodyweight [8, 11].

Cumulative fluid balance (CFB [liters]) corresponds to the difference between fluid input and fluid output. Fluid input includes all intravenous and enteral fluids. Fluid output includes urinary output, fecal losses, and evaporation as well as fluid losses via drains and nasogastric/orogastric tubes. Measurement of fluid in- and output started at the time of admission to the ICU.

CFB in relation to admission bodyweight was calculated using the following formula: ((cumulative fluid input [liters] – cumulative fluid output [liters]) / bodyweight [kg]) x 100 [5, 11, 12, 22, 23].

ICU day 3 was chosen for FA assessment because previous investigations indicate a strong relationship between mortality and fluid overload at ICU day 3 [8].

#### Acute kidney injury (AKI)

AKI and its stages were defined using the serum creatinine values in accordance to Kidney Disease Improving Global Outcomes (KDIGO) consensus criteria [24].

#### MAKE30

MAKE30 is a well-established composite outcome of death, need for new RRT of any duration, or prolonged kidney dysfunction within 30 days after ICU admission [5, 25–32].

Death was defined as all-cause mortality in the first 30 days after ICU admission.

Need for new RRT was defined as receipt of any modality of RRT of any duration until day 30 after ICU admission. We defined persistent renal dysfunction as a decline in estimated glomerular filtration rate (eGFR) of ≥ 25% and/or an increase in plasma creatinine level of ≥ 200% compared to the baseline [26, 29, 33].

Patients with RRT before ICU admission could only meet the MAKE30 endpoint if they died within 30 days, as they were not eligible to fulfil the other two components [27].

#### Past medical history

The data on the past medical history (history of CKD, chronic liver disease, cancer, and immune deficiency) was obtained from the minimal dataset of the Swiss Society of Intensive Care Medicine (MDSi) [34]. Key indicators from all Swiss ICUs are recorded in the MDSi, which are important for the certification of ICUs in Switzerland (quality management and benchmarking). The diagnoses in the MDSi are based on ICD-10 coding.

### Ethical consideration

The study was approved by the ethics committee of Bern University Hospital (EC no.: 2018 − 00436), who waived the need for individual informed consent due to the observational nature of the analysis.

### Statistical analysis

The statistical analyses were performed using IBM SPSS Statistics 28.0 and R (R Studio, PBC, Version 4.2.2).

Data is presented by mean and standard error or median and interquartile range (IQR).

To test for relationship, the chi-square test (categorical variables), the Mann-Whitney U test (ordinal and not-normally distributed continuous variables), and the independent-samples t-test (continuous variables) was used.

For continuous variables with missing values (APACHE, plasma creatinine at admission, and lactate levels at admission) multiple imputation was used. With patient characteristics as predictors, five imputations were created for each variable (see Supplemental Fig. 1).

To assess the association between FA and the primary / secondary endpoints first a univariate analysis using a binomial logistic regression (dichotomous dependent variables), or a linear regression (continuous dependent variables) was conducted. In a second step the model was adjusted for possible confounders using a multivariable model including all patient characteristics showing an association with FA. (Adjusted) odds ratio (OR / aOR) and coefficient β with 95% confidence intervals (CI) were reported to assess the influence of the independent variables. Confounders were defined as patient characteristics significantly associated with FA (age, APACHE, planned vs. emergency admission, creatinine at admission, history of CKD, history of chronic liver disease, history of immune deficiency).

To account for a possible influence of CKD and AKI on the results, we conducted a sensitivity analysis testing for an association between FA and the primary and secondary outcomes in each CKD group and KDIGO AKI stage separately.

In addition, we calculated the mean and standard error for serum creatinine levels during hospitalization in both groups: FA and no FA. These values were plotted over time for all patients and separately for AKI KDIGO stages and for the presence of CKD.

The natural log-transformed creatinine measurements were used as a response variable to fit a linear mixed model with an autoregressive covariance structure using the R package *glmmTMB* 1.1.7 [35] and its *ar1* function. Patient identities were included as random intercepts due to the necessity of grouping the creatinine measurements by patient. The autoregressive covariance structure defined by day groups over patient identities was included so to account for the interdependencies of the autocorrelated consecutive measurements over time. APACHE II scores at admission, patient age, and time of serum creatinine measurement (in days) were included as numerical fixed effects. FA category, immune deficiency, CKD, chronic liver disease, and admission type were included as categorical fixed effects. The family distribution was specified as “gaussian”, the model’s summary statistics including marginal mean estimates, relative changes, standard errors and p-values were computed with the R package *broom.mixed* 0.2.9.4 [36]. The effect size estimates presented in the table were exponentiated so to represent actual creatinine values in µmol/L and not their logarithmic values as specified in the model. The model’s QQ plots and residual plots were obtained with the *DHARMa* 0.4.6 R package [37]. The effect size plots show the model’s marginal means of each variable while fixing the confounders at their average values and were obtained with the R package *effects* 4.2-2 [38]. Consecutive models were further developed within AKI KDIGO stage and CKD categories to compare the model’s estimates across patient subgroups. One last further model was developed considering only the creatinine measurements during the first 3 days after admission.

## Results

Out of 19,476 patients screened, 13,326 patients met the inclusion criteria for analysis (see Fig. 1). The median age was 65 (IQR: 53–74) years, 8,536 (64.1%) participants were male. Known pre-existing kidney disease was present in 2,955 (22.2%) patients, 357 (2.7%) were on chronic dialysis before admission. Patient characteristics are shown in Table 1.

**Table 1:** Patient characteristics

	Total	FA at day 3	no FA at day 3	p-value
	13,326	1,100 (8.3)	12,226 (91.7)	
**Demographics**				
Male, n (%)	8,536 (64.1)	677 (61.5)	7,859 (64.3)	0.070
Age [years], median (IQR)	65 (53–74)	67 (56–74)	64 (53–73)	**< 0.001**
**Past medical history**				
History of CKD, n (%)	2,955 (22.2)	400 (36.4)	2,555 (20.9)	**< 0.001**
History of chronic liver disease, n (%)	1,594 (12.0)	218 (19.8)	1,376 (11.3)	**< 0.001**
History of cancer, n (%)	2,382 (17.9)	175 (15.9)	2,207 (18.1)	0.076
History of immune deficiency, n (%)	1,282 (9.6)	183 (16.6)	1,099 (9.0)	**< 0.001**
**ICU stay**				
APACHE II *, mean (Standard-Error)	15.30 (0.09)	20.30 (0.34)	14.85 (0.09)	**< 0.001**
Planned admission, n (%)	5,720 (42.9)	530 (48.2)	5,190 (42.5)	**< 0.001**
Creatinine at admission [µmol/ml] *, mean (Standard-Error)	112.70 (0.96)	155.94 (5.39)	108.81 (0.92)	**< 0.001**
Lactate levels at admission [mmol/l] *, mean (Standard-Error)	1.93 (0.01)	3.31 (0.08)	1.80 (0.01)	**< 0.001**
Vasopressors at admission, n (%)	383 (2.9)	63 (5.7)	320 (2.6)	**< 0.001**
Mechanical ventilation at admission, n (%)	4,070 (30.6)	555 (50.5)	3,515 (28.8)	**< 0.001**

Medians and interquartile range (IQR) or mean and standard deviation (SD) or total numbers (relative frequencies) are given. *FA*: fluid accumulation; *CKD*: chronic kidney disease; *KDIGO*: Kidney Disease: Improving Global Outcomes; *ICU*: intensive care unit; *APACHE II*: Acute Physiology and Chronic Health Evaluation II score; *ARDS*: acute respiratory distress syndrome

* Combined data after 5 imputations

Bold values represent a p-value < 0.05

A total of 1,100 (8.3%) patients had FA at ICU day 3. In the FA group the median degree of FA was 7.2% (IQR: 5.8–9.5) compared to 0.2% (-0.8–1.3) in patients without FA (*p* < 0.001). The median CFB at ICU day 3 was 5,267 mL (IQR: 4,167–6,994) in patients with FA and 124 mL (-670–1,033) in the non-FA group. 7,047 (52.9%) patients developed an AKI during the ICU stay (stage 1: 2,622 patients (19.7%); stage 2: 444 patients (3.3%); stage 3: 3,842 patients (28.8%)).

### Primary outcome

**Fig. 2:**
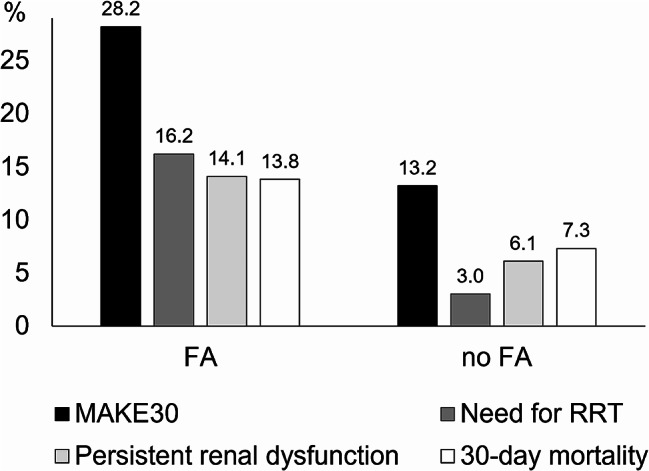
MAKE30 and its subcomponents in patients with and without FA. *FA*: fluid accumulation; *MAKE30*: major adverse kidney events in the first 30 days after intensive care unit (ICU) admission; *RRT*: renal replacement therapy

Out of the 1,100 patients with FA, 310 (28.2%) met the MAKE30 endpoint in comparison to 1,608 patients (13.2%) in the non-FA group (*p* < 0.001; see Fig. 2). FA at ICU day 3 was significantly associated with MAKE30 in the univariable (OR 2.59 [95%CI 2.25–2.99]) and multivariable model (aOR 1.96 [1.67–2.30], *p* < 0.001), see Table 2. This significant association also existed in all subgroups analyzed (see Supplemental Table 1).

**Table 2:** Primary and secondary outcomes

	Univariable model (unadjusted)		Multivariable model (adjusted *)	
	OR (95%-CI) / Regression-Co-efficient B (95%-CI)	p-value	OR (95%-CI) / Regression-Co-efficient B (95%-CI)	p-value
Primary endpoint				
MAKE30	2.59 (2.25–2.99)	**< 0.001**	1.96 (1.67–2.30)	**< 0.001**
**Secondary endpoints**				
**Need for RRT**	6.27 (5.18–7.60)	**< 0.001**	3.83 (3.02–4.84)	**< 0.001**
Duration of RRT on the ICU [days]	*3.41 (2.06–4.76)*	***< 0.001***	*1.48 (1.14–1.82)*	***< 0.001***
Acute kidney injury (AKI)	2.18 (1.91–2.49)	**< 0.001**	1.76 (1.53–2.03)	**< 0.001**
KDIGO stage 1	0.88 (0.75–1.03)	0.119		
KDIGO stage 2	0.65 (0.43–0.98)	**0.039**	0.60 (0.39–0.91)	**0.015**
KDIGO stage 3	2.58 (2.28–2.92)	**< 0.001**	2.01 (1.76–2.31)	**< 0.001**
**Persistent renal dysfunction** ^+^	2.54 (2.10–3.08)	**< 0.001**	1.72 (1.40–2.12)	**< 0.001**
**30-day mortality**	2.04 (1.69–2.45)	**< 0.001**	1.70 (1.38–2.09)	**< 0.001**

Medians and interquartile range (IQR) or odds ratio (OR) and 95% confidence interval (95%-CI) or regression coefficient (95%-CI) are given. *FA*: fluid accumulation; *MAKE30*: major adverse kidney events in the first 30 days after ICU admission; *RRT*: renal replacement therapy; *ICU*: intensive care unit; *AKI*: acute kidney injury; *KDIGO*: Kidney Disease: Improving Global Outcomes

^+^ only patients without chronic dialysis before admission (*n* = 12,749, therof 1,012 with FA)

* The multivariable regression model is adjusted for age, APACHE II Score, type of admission, creatinine at admission, history of chronic kidney disease (CKD), history of liver disease, and history of immune deficiency.Bold values represent a p-value < 0.05

### Secondary outcomes

FA was significantly associated with all individual components of the MAKE30 composite endpoint after adjustment (need for RRT: aOR 3.83 [95%CI 3.02–4.84]; persistent renal dysfunction: aOR 1.72 [1.40–2.12]; 30-day mortality: aOR 1.70 [1.38–2.09]; all *p* < 0.001), see Table 2.

**Fig. 3:**
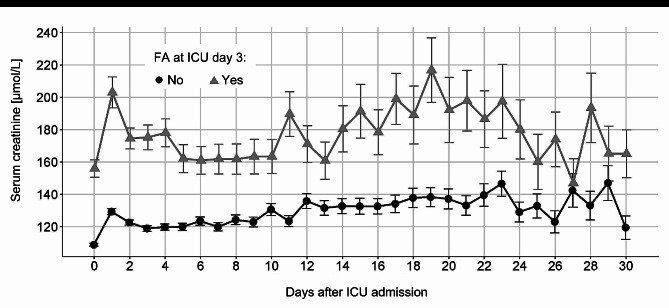
Serum creatinine trajectories in the first 30 days for patients with and without FA. *FA*: fluid accumulation; *ICU*: intensive care unit

The creatinine trajectories of the FA and non-FA group are shown in Fig. 3, with higher non-adjusted serum creatinine values in the FA group than in the non-FA group during the 30-day observational period. The trajectories according to the AKI KDIGO stages as well as the presence of CKD are shown in Supplemental Fig. 2.

The autoregressive linear mixed model identified FA as a significant independent predictor for the creatinine trajectory in the first three days of ICU stay (*p* < 0.001), see Table 3 and Supplemental Fig. 3.

**Table 3:** Full adjusted autoregressive linear mixed model for serum creatinine values in the first 3 days

Variable	Effect estimate (95%-CI)	p value
FA at ICU day 3	1.14 (1.11–1.17)	**< 0.001**
Age	1.00 (1.00–1.00)	**0.001**
Chronic kidney disease	1.86 (1.83–1.90)	**< 0.001**
Chronic liver disease	1.08 (1.06–1.11)	**< 0.001**
Immune deficiency	1.19 (1.16–1.22)	**< 0.001**
Emergency admission (vs. planned)	1.01 (1.00–1.03)	0.061
APACHE II	1.01 (1.01–1.01)	**< 0.001**
Day 1	1.03 (1.03–1.04)	**< 0.001**
Day 2	0.99 (0.98–1.00)	0.156
Day 3	0.93 (0.92–0.94)	**< 0.001**
Intercept	66.24 (64.11–68.44)	**< 0.001**

Effect estimates and 95% confidence interval (95%-CI) are given. *FA*: fluid accumulation;* ICU*: intensive care unit; *APACHE II*: Acute Physiology and Chronic Health Evaluation II score

Bold values represent a p-value < 0.05

### Sensitivity analysis

The impact of FA on MAKE30 and its individual components in relationship to the stage of AKI is shown in Supplemental Table 2. FA was only independently associated with MAKE30 in patients with an AKI stage 3. Patients with FA and an AKI stage 3 had the highest percentage of FA at day 3 (median: 7.8% [IQR 6.2–10.7]) as well as the highest maximal FA at any time point (median: 8.5% [IQR 6.4–11.7]) compared to stage 1 AKI (6.9% [IQR 5.8–9.0); 7.6% [6.1–10.0]) and non-AKI patients (6.4% [IQR 5.5–8.6]; 6.8% [IQR 5.7–9.2]).

FA was associated with MAKE30 and all individual components regardless of the presence or absence of CKD (see Supplemental Table 3).

The autoregressive linear mixed model indicates that FA at ICU day 3 and immune deficiency were the strongest predictors for serum creatinine trajectory during the observation period in both, patients with and without pre-existing CKD. (Table 4 and Supplemental Fig. 3). Supplemental Tables 4 and 5 as well as Supplemental Fig. 4 show the analyses in subgroups according to the AKI KDIGO stages and the presence of CKD, respectively.

**Table 4:** Full adjusted autoregressive linear mixed model for serum creatinine values in the first 30 days

Variable	Effect estimate (95%-CI)	p value
FA at ICU day 3	1.11 (1.08–1.14)	**< 0.001**
Age	1.00 (1.00–1.00)	**< 0.001**
Chronic kidney disease	1.82 (1.78–1.85)	**< 0.001**
Chronic liver disease	1.08 (1.06–1.11)	**< 0.001**
Immune deficiency	1.19 (1.16–1.22)	**< 0.001**
Emergency admission (vs. planned)	1.01 (1.00–1.03)	0.115
APACHE II	1.01 (1.01–1.01)	**< 0.001**
Day	0.99 (0.99–0.99)	**< 0.001**
Intercept	63.64 (61.62–65.71)	**< 0.001**

Effect estimates and 95% confidence interval (95%-CI) are given. *FA*: fluid accumulation; *ICU*: intensive care unit; *APACHE II*: Acute Physiology and Chronic Health Evaluation II score

Bold values represent a p-value < 0.05

## Discussion

This observational cohort study indicates that FA in the critically ill is independently associated with MAKE30 and its sub-components. FA substantially and independently influences mid-term creatinine trajectory over 30 days after ICU admission. This seems especially true in patients with AKI stage 3 during ICU stay and is independent of the presence of CKD.

Our findings go in-line with several other studies analyzing specific subgroups of ICU patients.

In a recent multicenter observational study, a higher cumulative fluid balance at ICU day 3 was associated with MAKE30 in septic patients [29]. Another retrospective cohort study analyzing 863 patients admitted to the ICU with AKI revealed that patients developing FA (> 5% or > 10%) during the first five ICU days were less likely to experience renal recovery (defined as creatinine values below 150% of baseline and five consecutive days without RRT in the first 28 days after ICU admission) [7]. A systematic review and meta-analysis of Zhang et al. summarized four cohort studies regarding the association of FA with renal recovery in patients with AKI. Although there was a trend of reduced kidney recovery in patients with FA, the data were insufficient to demonstrate a clear relationship [17].

Two other studies analyzed critically ill patients requiring RRT for AKI. The first, a recent retrospective cohort study, showed higher aOR for MAKE90 in patients with a FA > 10% of bodyweight [5]. In the second analysis by Heung et al. a higher degree of FA at the time of RRT initiation resulted in poorer recovery of renal function within one year (discontinuation from dialysis for at least 2 weeks) [39].

Additional data supporting these findings are provided by pediatric studies. In a recent study with 1,017 critically ill children, a greater peak FA was associated with MAKE30 [30]. Another study involving children requiring RRT showed that survivors with a FA of > 20% had a significantly longer time to renal recovery (26 vs. 8 days, *p* = 0.0038) [40].

In contrast to our results, a single-center study with 18,084 ICU patients who received RRT found no association between a negative or positive fluid balance prior to initiation of RRT and renal recovery (defined as alive and without the need of RRT one year after admission) [22]. In this study, however, a positive fluid balance was defined as a FA ≥ 0% in contrast to the used cut-off of 5% or 10% in most other studies. This might be a source of considerable bias, as an only slightly positive FB does not necessarily have a negative impact on organ function and thus may not be equivalent to FA with organ function or a higher degree of fluid accumulation.

In summary, there are several studies showing an association between FA and mid- to long-term renal outcome. However, these studies mostly examine specific patient groups such as patients with AKI, patients with septic shock, patients requiring RRT or children.

In our exploratory analysis, we found a strong association between FA at ICU day 3 and higher serum creatinine trajectory in the first three days after ICU admission. In addition, the negative effects of FA on short and mid-term renal outcome are mainly present in patients with severe acute kidney injury (AKI 3) and were without relation to prior CKD status in our analysis. This goes in line with the recently published multicenter, observational EPIS-AKI study: patients developing a postsurgical AKI received intra- and postoperatively significantly more fluids compared to patients without AKI [41]. In addition, a postsurgical AKI was strongly associated with MAKE90, with the highest incidence in patients with AKI stage 3 [41]. Furthermore, Bouchard et al. analyzed 542 ICU patients with diagnosis of AKI who were enrolled in a prospective multicenter observational study [23]. In this trial, FA (> 10%) at the time of AKI diagnosis was associated with impaired recovery of renal function (defined as serum creatinine level ≤ 20% or ≤ 44 µmol/l above the baseline value). Additionally, kidney recovery was less likely in patients with FA at the time of serum creatinine peak [23]. In line with our results, this may indicate that FA in the presence of AKI has more of an impact on renal recovery than FA before the onset of AKI. Thus, patients with FA and severe AKI should be considered especially “at risk” for a delayed or no renal recovery that is associated with end-stage renal disease and dialysis-dependency [32, 42, 43] as well as higher mortality [32, 42–44].

However, whether therapeutic intervention in patients “at risk” would improve non-kidney outcome is currently uncertain. The recently published STARRT-AKI trial showed that an accelerated renal-replacement strategy in 2,927 patients with stage 2 or 3 AKI had no influence on 90-day mortality or MAKE90. In contrast, more adverse effects occurred, and a greater proportion of patients required RRT after 90 days in the accelerated strategy group [45]. In addition, in a secondary analysis of the STARRT-AKI trial, an accelerated initiation of RRT reduced the median CFB in the 14 days following randomization by 1,137 ml but had no impact on the 90-day-mortality [46]. Hospital-free days were greater in patients with FA > 10% with the accelerated strategy. Older observational studies indicated an association between an early initiation of RRT and a reduced 60- or 90-day mortality, respectively [47–49].

These findings are supported by two other multicenter, randomized trials showing no association with an accelerated renal-replacement strategy and 60- and 90-day mortality, respectively, in patients with severe AKI [50, 51].

### Limitations

Several limitations of our investigation warrant discussion.

First, because of the retrospective, observational and monocentric study design, our investigations are of exploratory nature and all limitations typical for this study design apply.

Second, due to the retrospective nature of the study there was incomplete data. We tried to address this by performing imputations, but this may still be a source of bias. Furthermore, creatinine values before admission were not available. This may have resulted in a too low AKI classification for some patients in case of an already elevated serum creatinine at admission.

Third, we have no data for fluid in- and output before ICU admission, e.g., in the emergency department or during surgery. In addition, we could not account for insensible fluid losses such as diarrhea or perspiration. This may both have affected the calculation of the CFB.

Fourth, there is currently no uniform definition of FA, and all approaches have their drawbacks. FA represents a continuum, and it remains unclear beyond which point FA negatively affects patient outcomes. We chose a cut-off of 5% in accordance with current literature [8, 11] but we cannot exclude that the choice of a different cut-off or a continuous scale would have led to other results. Further, we would like to acknowledge that FA with organ-failure is the driver behind the increased mortality and morbidity associated with FA and that there is no universally agreement upon time point of FA assessment.

Fifth, some patients were admitted to the IMC being critically ill but with a lower disease severity than the ICU patients. Considering only ICU patients could have resulted in a higher incidence of FA and AKI.

Sixth, the multivariable regression analyses and the autoregressive linear mixed models were adjusted for numerous confounders. However, the renal function is likely to be affected by other confounders that were not available and therefore not considered (e.g., cardiac function, the occurrence of infection/sepsis or administered, potentially nephrotoxic drugs).

Finally, since no urine output values were available for our patient cohort, AKI definition was based on creatinine values alone. However, a recent study in patients with sepsis-associated AKI showed a better prognosis regarding renal recovery and mortality in patients with AKI diagnosed by low urine output alone [32].

## Conclusions

In our observational cohort study, FA was independently associated with MAKE30 in a mixed cohort of critically ill patients. This association was independent from pre-existing CKD and strongest in patients with AKI stage 3. FA substantially and independently influences short- and mid-term creatinine trajectory over 3 or 30 days after ICU admission. This may hint towards FA being a major risk factor for reduced renal recovery and patients with FA, especially those with severe acute kidney should be considered “at risk” for renal recovery. Its therapeutic relevance for kidney-independent ICU outcomes remains unclear. Further high-quality investigations are needed.

### Electronic supplementary material

Below is the link to the electronic supplementary material.

Supplementary Material 1

## Data Availability

The datasets are available from the corresponding author on reasonable request.

## Abbreviations

AKI: Acute kidney injury
APACHE II: Acute Physiology and Chronic Health Evaluation II score
ARDS: Acute respiratory distress syndrome
CFB: Cumulative fluid balance
CKD: Chronic kidney disease
eGFR: Estimated glomerular filtration rate
FA: Fluid accumulation
IMC: Intermediate care unit
ICU: Intensive care unit
KDIGO: Kidney Disease Improving Global Outcomes
MAKE30: Major adverse kidney events in the first 30 days after ICU admission
RRT: Renal replacement therapy
