# Supplementary material for: Influence of fluid accumulation on major adverse kidney events in critically ill patients – an observational cohort study
Source: Ann Intensive Care. 2024 Apr 8;14:52. doi: 10.1186/s13613-024-01281-7 (PMC11001812; doi:10.1186/s13613-024-01281-7)
Supplement: Supplementary file 1 — Supplementary Material 1 [file 13613_2024_1281_MOESM1_ESM.docx]

***Supplemental content to***

**Influence of fluid accumulation on major adverse kidney events in critically ill patients – an observational cohort study**

Debora M. Hofer, Livio Ruzzante, Jan Waskowski, Anna S. Messmer, Carmen A. Pfortmueller

Department of Intensive Care Medicine, Inselspital, Bern University Hospital, Bern, Switzerland

DH: [debora.hofer@extern.insel.ch](mailto:debora.hofer@extern.insel.ch)

LR: [livio.ruzzante@gmail.com](mailto:livio.ruzzante@gmail.com)

JW: [jan.waskowski@insel.ch](mailto:jan.waskowski@insel.ch)

ASM: [anna.messmer@insel.ch](mailto:anna.messmer@insel.ch)

CAP: [carmen.pfortmueller@insel.ch](mailto:carmen.pfortmueller@insel.ch)

**Address for correspondence:**

Debora Hofer; MD

Department of Intensive Care Medicine

Inselspital, Bern University Hospital

Freiburgstrasse 18

CH-3010 Bern, Switzerland

[debora.hofer@extern.insel.ch](mailto:debora.hofer@extern.insel.ch)

## Table of contents

[**Supplemental Figure 1**: Imputation diagrams 3](#_Toc142492761)

**Supplemental Table 1**: Association of FA at ICU day 3 and MAKE30 in different
subgroups 4

[**Supplemental Figure 2**: Serum creatinine trajectories for the first 30 days after
ICU admission presented by (A) CKD and (B) AKI subgroups 4](#_Toc142492762)

[**Supplemental Figure 3**: Full adjusted autoregressive linear mixed models showing
the effect of different variables on serum creatinine values during (A) the first 30 days of hospitalization and (B) the first 3 days after admission 6](#_Toc142492763)

[**Supplemental Table 2**: Primary and secondary outcomes according to the presence
and stages of AKI 7](#_Toc142492764)

[**Supplemental Table 3**: Primary and secondary outcomes according to the presence
of CKD 8](#_Toc142492765)

[**Supplemental Table 4**: Full adjusted autoregressive linear mixed model for serum
creatinine values in the first 30 days after ICU admission according to the presence
of CKD 9](#_Toc142492766)

[**Supplemental Table 5**: Full adjusted autoregressive linear mixed model for serum
creatinine values in the first 30 days after ICU admission for the different AKI stages 10](#_Toc142492767)

[**Supplemental Figure 4**: Full adjusted autoregressive linear mixed models showing
the effect of different variables on serum creatinine values during the first 30 days of hospitalization for the different (A) CKD and (B) AKI stage subgroups 11](#_Toc142492768)

## Supplemental Figure 1: Imputation diagrams


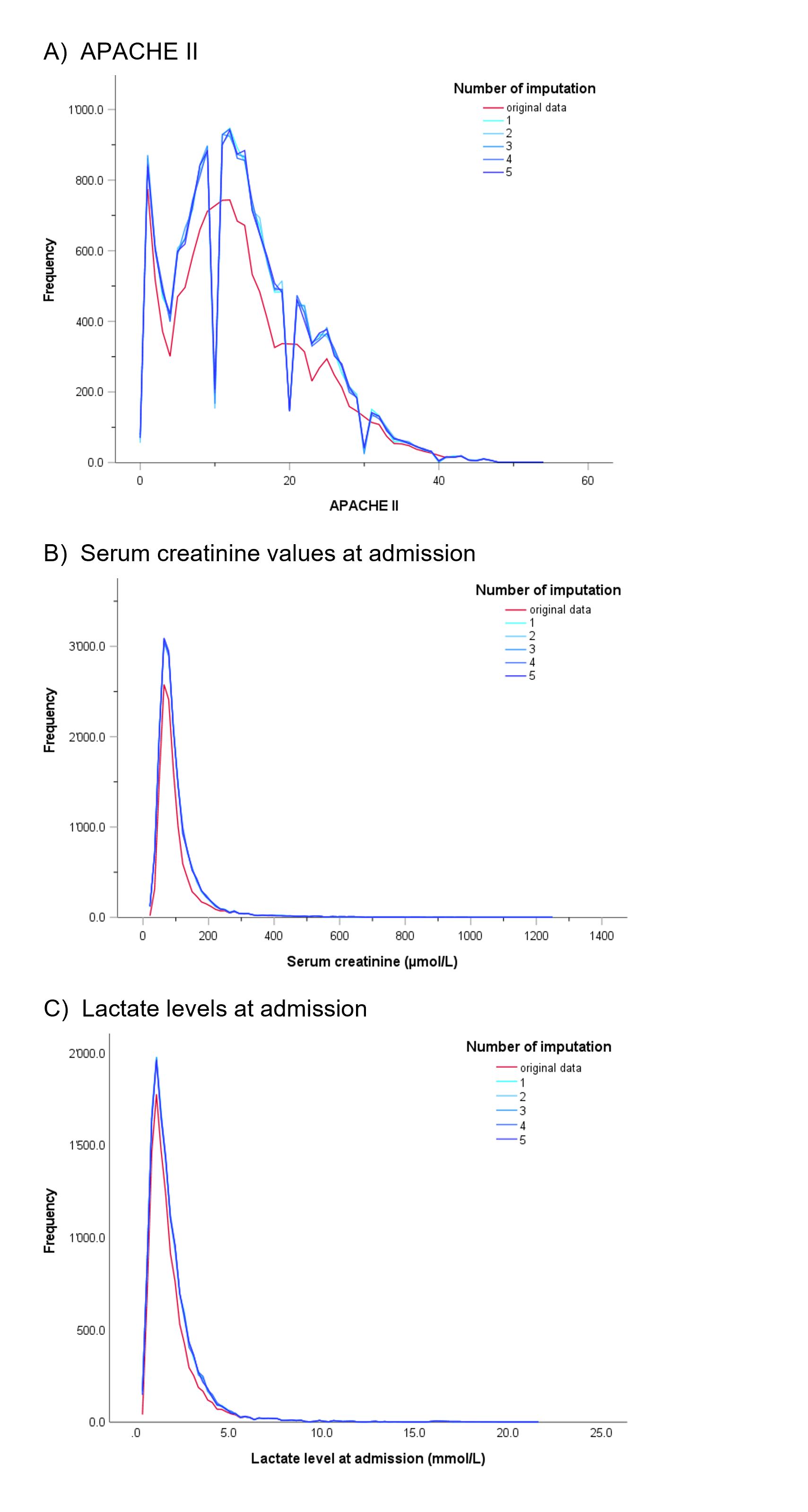


Imputation diagrams for the three imputed variables. APACHE II: Acute Physiology and Chronic Health Evaluation II score

## Supplemental Table 1: Association of FA at ICU day 3 and MAKE30 in different patient subgroups

| **Patient subgroup** | **Incidence of MAKE30** | **Univariable model (unadjusted)** | | **Multivariable model (adjusted °)** | |
| --- | --- | --- | --- | --- | --- |
|  |  | **OR (95%-CI)** | **p-value** | **OR (95%-CI)** | **p-value** |
| Sepsis / septic shock ^+^ n=1,783 (13.4) | 532 (29.8) | 2.88 (2.25 - 3.69) | <0.001 | 2.42 (1.86 - 3.14) | <0.001 |
| Severe heart failure / cardiogenic shock ^+^ n=4,101 (30.8) | 816 (19.9) | 2.27 (1.84 - 2.79) | <0.001 | 2.13 (1.69 - 2.69) | <0.001 |
| Respiratory failure / ARDS ^+^ n=1,822 (13.7) | 532 (29.2) | 2.79 (2.07 - 3.76) | <0.001 | 2.43 (1.76 - 3.36) | <0.001 |
| Non traumatic neurological disease ^+^ n=3,214 (24.1) | 470 (14.6) | 3.32 (1.75 - 6.29) | <0.001 | 2.56 (1.29 - 5.10) | <0.001 |
| Surgery prior to admission / major trauma ^+^ n=6,110 (45.9) | 619 (10.1) | 2.55 (2.08 - 3.14) | <0.001 | 1.85 (1.48 - 2.31) | <0.001 |
| Admission to the ICU n=7,037 (52.8%) | 1'324 (18.8) | 2.42 (2.06 - 2.86) | <0.001 | 2.13 (1.77 - 2.56) | <0.001 |
| Admission to the IMC n=6,289 (47.2%) | 594 (9.4) | 1.99 (1.47 - 2.69) | <0.001 | 1.47 (1.06 - 2.04) | 0.022 |

Total numbers (relative frequencies) or odds ratio (OR) and 95% confidence interval (95%-CI) are given.
FA: Fluid accumulation at ICU day 3; MAKE30: Major adverse kidney events in the first 30 days after ICU admission; ICU: Intensive care unit; IMC: Intermediate care unit.
^+^ Patients may be assigned to one or more subgroups based on their discharge diagnosis.
° The multivariable regression model is adjusted for age, APACHE II Score, type of admission, creatinine at admission, history of chronic kidney disease (CKD), history of liver disease, and history of immune deficiency.

## Supplemental Figure 2: Serum creatinine trajectories for the first 30 days after ICU admission presented by (A) CKD and (B) AKI subgroups


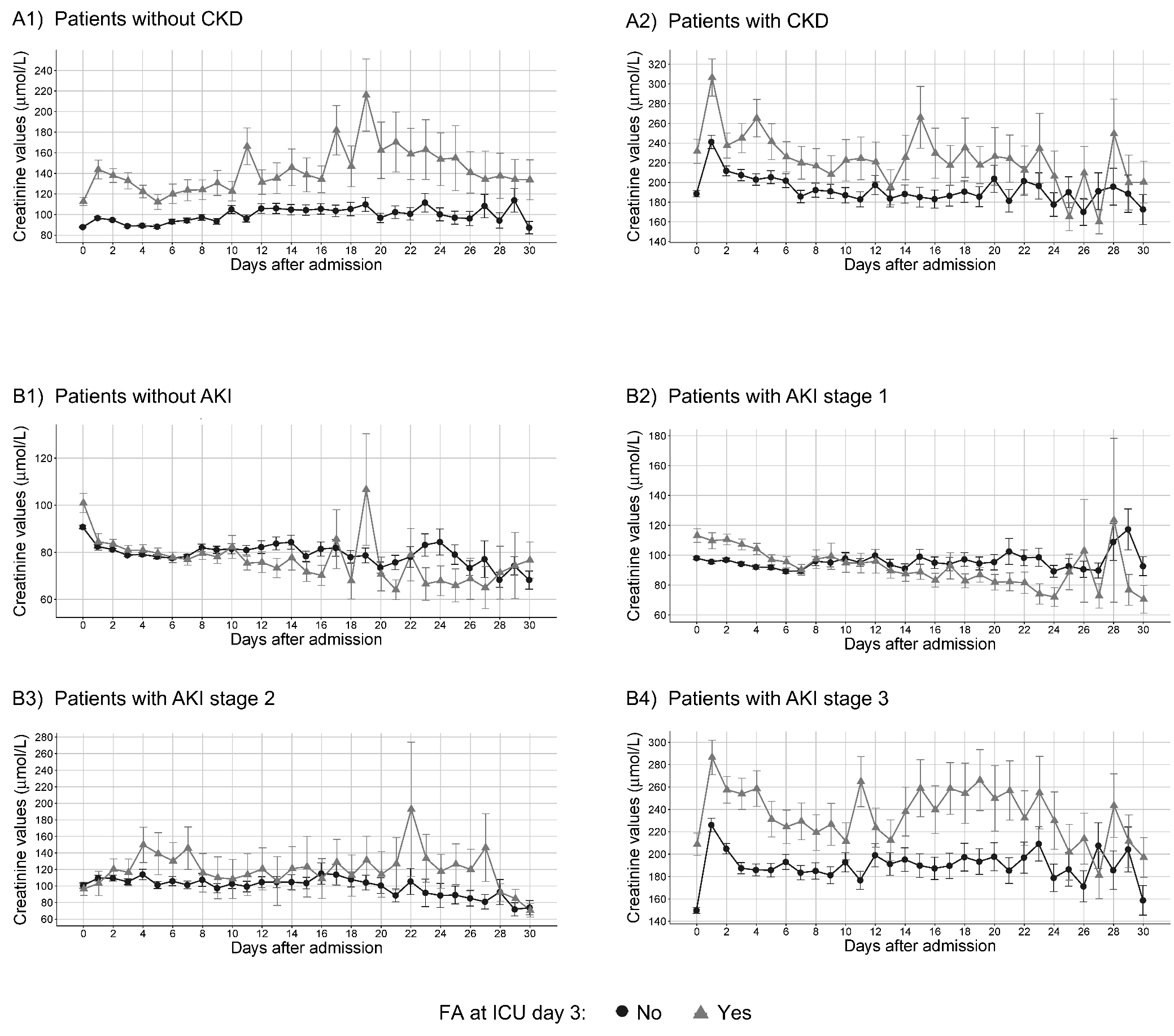


ICU: intensive care unit; CKD: chronic kidney disease; AKI: acute kidney injury; FA: fluid accumulation

## Supplemental Figure 3: Full adjusted autoregressive linear mixed models showing the effect of different variables on serum creatinine values during (A) the first 30 days of hospitalization and (B) the first 3 days after admission


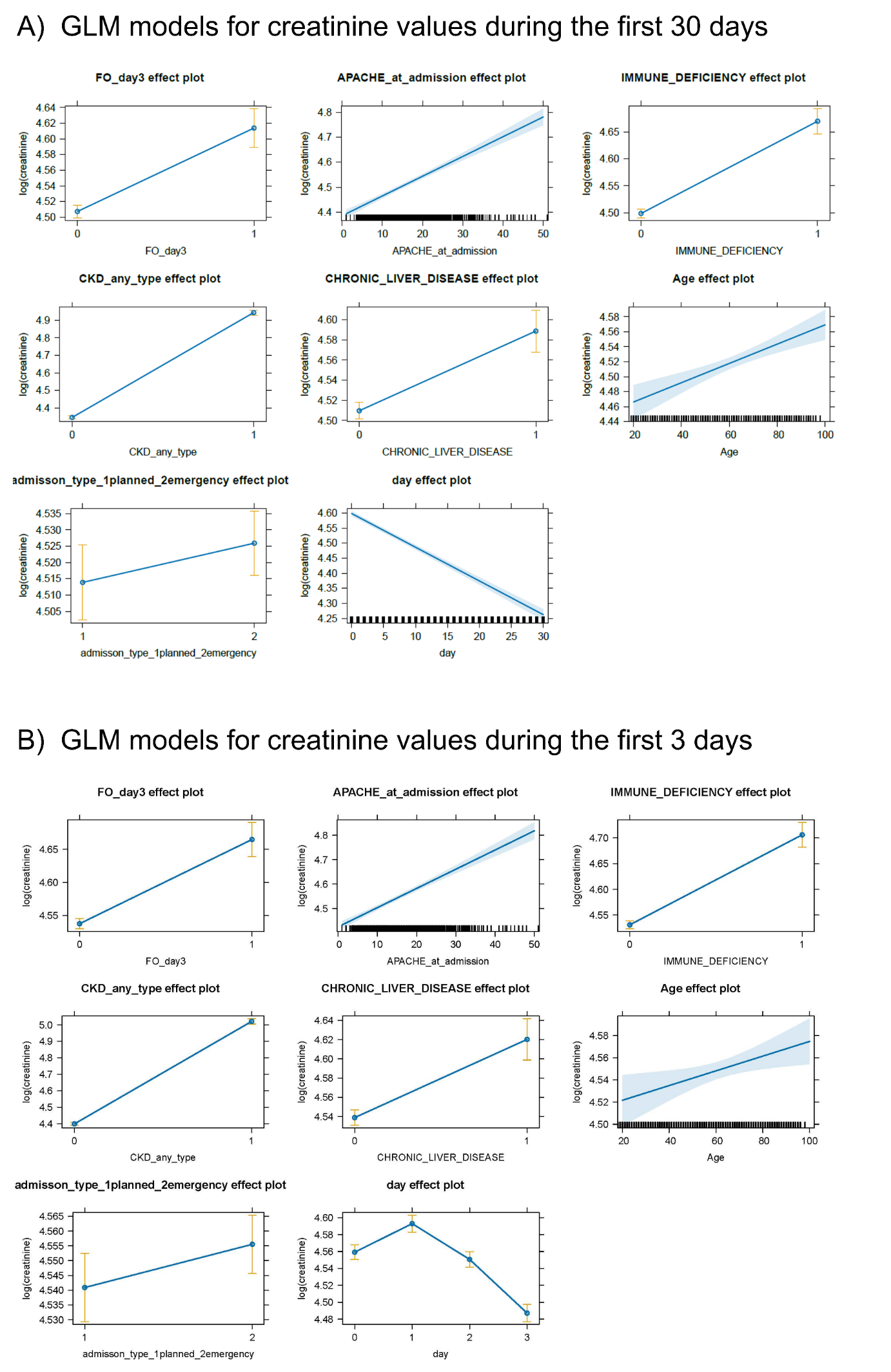


The black bars density along the horizontal axis represents the frequencies of the values in the data, the dark blue line the relative change between the intercept and the alternative categories across effects, while the light blue area (for numeric fixed effects) and the yellow bars (for categorical fixed effects) indicate the 95% confidence intervals of the model estimates.
FA: fluid accumulation; APACHE II: Acute Physiology and Chronic Health Evaluation II score; CKD: chronic kidney disease.

## Supplemental Table 2: Primary and secondary outcomes according to the presence and stages of AKI

|  | **Univariable model (unadjusted)** | | **Multivariable model (adjusted*)** | |
| --- | --- | --- | --- | --- |
|  | OR / *regression co-efficient B* (95%-CI) | p-value | OR / *regression co-efficient B* (95%-CI*)* | p-value |
| **Patients without AKI** (n=6,279) | | | | |
| **Primary endpoint** |  |  |  |  |
| MAKE30 | 1.38 (0.96 - 2.00) | 0.084 |  |  |
| **Secondary endpoints** |  |  |  |  |
| Persistent renal dysfunction ^+^ | 1.79 (0.98 - 3.28) | 0.059 |  |  |
| Mortality after 30 days | 1.24 (0.81 - 1.91) | 0.323 |  |  |
|  |  |  |  |  |
| **Patients with AKI KDIGO stage 1** (n=2,622) | | | | |
| **Primary endpoint** |  |  |  |  |
| MAKE30 | 1.49 (0.99 - 2.24) | 0.058 |  |  |
| **Secondary endpoints** |  |  |  |  |
| Persistent renal dysfunction ^+^ | 2.73 (1.66 - 4.47) | **<0.001** | 2.07 (1.23 - 3.50) | **0.006** |
| Mortality after 30 days | 1.04 (0.60 - 1.80) | 0.887 |  |  |
|  |  |  |  |  |
| **Patients with AKI KDIGO stage 2** (n=444) | | | | |
| **Primary endpoint** |  |  |  |  |
| MAKE30 | 1.75 (0.67 - 4.56) | 0.250 |  |  |
| **Secondary endpoints** |  |  |  |  |
| Persistent renal dysfunction ^+^ | 1.25 (0.36 - 4.35) | 0.729 |  |  |
| Mortality after 30 days | 2.76 (0.88 - 8.61) | 0.081 |  |  |
|  |  |  |  |  |
| **Patients with AKI KDIGO stage 3** (n=3,842) | | | | |
| **Primary endpoint** |  |  |  |  |
| MAKE30 | 2.42 (2.01 - 2.92) | **<0.001** | 1.95 (1.59 - 2.39) | **<0.001** |
| **Secondary endpoints** |  |  |  |  |
| Need for RRT | 3.97 (3.22 - 4.90) | **<0.001** | 2.85 (2.21 - 3.66) | **<0.001** |
| Duration of RRT on the ICU | *4.30 (3.52 - 5.07)* | **<0.001** | *2.74 (1.36 - 4.11)* | **<0.001** |
| Persistent renal dysfunction ^+^ | 1.57 (1.24 - 1.99) | **<0.001** | 1.42 (1.11 - 1.82) | **0.006** |
| Mortality after 30 days | 2.26 (1.78 - 2.87) | **<0.001** | 1.88 (1.44 - 2.46) | **<0.001** |

Odds ratio (OR) and 95% coinfidence interval (95%-CI) or regression coefficient (95%-CI) are given.
MAKE30: major adverse kidney events in the first 30 days after ICU admission; RRT: renal replacement therapy; ICU: intensive care unit; AKI: acute kidney injury.
^+^ only patients without chronic dialysis before admission. * The multivariable regression model is adjusted for age, APACHE II Score, type of admission, creatinine at admission, history of chronic kidney disease (CKD), history of liver disease, and history of immune deficiency.
Bold values represent a p-value < 0.05.

## Supplemental Table 3: Primary and secondary outcomes according to the presence of CKD

|  | **Univariable model (unadjusted)** | | **Multivariable model (adjusted*)** | |
| --- | --- | --- | --- | --- |
|  | OR / *regression co-efficient B* (95%-CI) | p-value | OR / *regression co-efficient B* (95%-CI) | p-value |
| **Patients without CKD** (n=10,371) | | | | |
| **Primary endpoint** |  |  |  |  |
| MAKE30 | 2.89 (2.41 - 3.46) | **<0.001** | 2.05 (1.66 - 2.52) | **<0.001** |
| **Secondary endpoints** |  |  |  |  |
| Need for RRT | 9.88 (7.46 - 13.08) | **<0.001** | 4.98 (3.51 - 7.05) | **<0.001** |
| Duration of RRT on the ICU | *3.10 (0.50 - 5.69)* | ***0.020*** | *2.81 (0.17 - 5.45)* | ***0.037*** |
| Persistent renal dysfunction ^+^ | 2.86 (2.24 - 3.66) | **<0.001** | 1.86 (1.41 - 2.44) | **<0.001** |
| Mortality after 30 days | 2.30 (1.83 - 2.90) | **<0.001** | 1.87 (1.43 - 2.43) | **<0.001** |
|  |  |  |  |  |
| **Patients with CKD** (n=2,955) | | | | |
| **Primary endpoint** |  |  |  |  |
| MAKE30 | 1.69 (1.34 - 2.13) | **<0.001** | 1.68 (1.31 - 2.15) | **<0.001** |
| **Secondary endpoints** |  |  |  |  |
| Need for RRT | 3.00 (2.29 - 3.94) | **<0.001** | 2.72 (1.96 - 3.77) | **<0.001** |
| Duration of RRT on the ICU | *3.08 (1.77 - 4.39)* | ***<0.001*** | *2.33 (0.98 - 3.68)* | ***<0.001*** |
| Persistent renal dysfunction ^+^ | 1.42 (1.05 - 1.93) | **0.024** | 1.38 (1.01 - 1.89) | **0.046** |
| Mortality after 30 days | 1.38 (1.01 - 1.87) | **0.042** | 1.45 (1.04 - 2.02) | **0.03** |

Odds ratio (OR) and 95% coinfidence interval (95%-CI) or regression coefficient (95%-CI) are given.
CKD: chronic kidney disease; MAKE30: major adverse kidney events in the first 30 days after ICU admission; RRT: renal replacement therapy; ICU: intensive care unit.
^+^ only patients without chronic dialysis before admission. * The multivariable regression model is adjusted for age, APACHE II Score, type of admission, creatinine at admission, history of chronic kidney disease (CKD), history of liver disease, and history of immune deficiency.
Bold values represent a p-value < 0.05.

## Supplemental Table 4: Full adjusted autoregressive linear mixed model for serum creatinine values in the first 30 days after ICU admission according to the presence of CKD

| **Variable** | **Effect estimate (95%-CI)** | **p value** |
| --- | --- | --- |
| **Patients without CKD** | | |
| FA at ICU day 3 | 1.13 (1.10 – 1.16) | **<0.001** |
| Age | 1.00 (1.00 – 1.00) | **<0.001** |
| Chronic liver disease | 1.09 (1.07 – 1.12) | **<0.001** |
| Immune deficiency | 1.14 (1.10 – 1.17) | **<0.001** |
| Emergency admission (vs. planned) | 1.00 (0.99 – 1.02) | 0.540 |
| APACHE II | 1.01 (1.01 – 1.01) | **<0.001** |
| Day | 0.99 (0.99 – 0.99) | **<0.001** |
| Intercept | 59.69 (57.86 – 61.57) | **<0.001** |
| **Patients with CKD** | | |
| FA at ICU day 3 | 1.08 (1.02 – 1.14) | **0.009** |
| Age | 0.99 (0.99 – 0.99) | **<0.001** |
| Chronic liver disease | 1.04 (0.98 – 1.10) | 0.173 |
| Immune deficiency | 1.23 (1.16 – 1.30) | **<0.001** |
| Emergency admission (vs. planned) | 1.06 (1.02 – 1.10) | **0.008** |
| APACHE II | 1.01 (1.01 – 1.01) | **<0.001** |
| Day | 0.99 (0.99 – 0.99) | **<0.001** |
| Intercept | 164.23 (146.41 – 184.22) | **<0.001** |

Effect estimates and 95% confidence interval (95%-CI) are given.
CKD: chronic kidney disease; ICU: intensive care unit; FA: fluid accumulation; APACHE II: Acute Physiology and Chronic Health Evaluation II score. Bold values represent a p-value < 0.05.

## Supplemental Table 5: Full adjusted autoregressive linear mixed model for serum creatinine values in the first 30 days after ICU admission for the different AKI stages

| **Variable** | **Effect estimate (95%-CI)** | **p value** |
| --- | --- | --- |
| **Patients without AKI** | | |
| FA at ICU day 3 | 0.96 (0.93 – 0.99) | **0.029** |
| Age | 1.00 (1.00 – 1.00) | **<0.001** |
| Chronic kidney disease | 1.58 (1.55 – 1.62) | **<0.001** |
| Chronic liver disease | 1.01 (0.99 – 1.04) | 0.380 |
| Immune deficiency | 1.09 (1.06 – 1.12) | **<0.001** |
| Emergency admission (vs. planned) | 0.97 (0.95 – 0.98) | 0.540 |
| APACHE II | 1.00 (1.00 – 1.00) | **0.002** |
| Day | 0.99 (0.99 – 0.99) | **<0.001** |
| Intercept | 63.07 (61.18 – 65.05) | **<0.001** |
| **Patients with AKI KDIGO stage 1** | | |
| FA at ICU day 3 | 1.03 (0.98 – 1.07) | 0.263 |
| Age | 1.00 (1.00 – 1.00) | **<0.001** |
| Chronic kidney disease | 1.56 (1.51 – 1.61) | **<0.001** |
| Chronic liver disease | 1.03 (0.99 – 1.07) | 0.170 |
| Immune deficiency | 1.16 (1.11 – 1.21) | **<0.001** |
| Emergency admission (vs. planned) | 0.98 (0.95 – 1.00) | 0.952 |
| APACHE II | 1.00 (1.00 – 1.01) | **<0.001** |
| Day | 0.99 (0.99 – 0.99) | **<0.001** |
| Intercept | 63.47 (60.02 – 67.11) | **<0.001** |
| **Patients with AKI KDIGO stage 2** | | |
| FA at ICU day 3 | 1.06 (0.92 – 1.21) | 0.423 |
| Age | 1.00 (1.00 – 1.01) | **0.002** |
| Chronic kidney disease | 1.59 (1.47 – 1.72) | **<0.001** |
| Chronic liver disease | 1.08 (0.98 – 1.19) | 0.139 |
| Immune deficiency | 1.07 (0.97 – 1.18) | 0.183 |
| Emergency admission (vs. planned) | 1.00 (0.94 – 1.07) | 0.946 |
| APACHE II | 1.00 (0.99 – 1.01) | 0.214 |
| Day | 0.99 (0.99 – 0.99) | **<0.001** |
| Intercept | 60.80 (52.29 – 70.69) | **<0.001** |
| **Patients with AKI KDIGO stage 3** | | |
| FA at ICU day 3 | 1.11 (1.06 – 1.17) | **<0.001** |
| Age | 1.00 (0.99 – 1.00) | 0.247 |
| Chronic kidney disease | 1.97 (1.90 – 2.05) | **<0.001** |
| Chronic liver disease | 1.04 (0.99 – 1.09) | 0.101 |
| Immune deficiency | 1.23 (1.17 – 1.29) | **<0.001** |
| Emergency admission (vs. planned) | 1.01 (0.98 – 1.05) | 0.458 |
| APACHE II | 1.01 (1.01 – 1.01) | **<0.001** |
| Day | 0.99 (0.99 – 0.99) | **<0.001** |
| Intercept | 80.21 (73.79 – 87.18) | **<0.001** |

Effect estimates and 95% confidence interval (95%-CI) are given.
ICU: intensive care unit; AKI: acute kidney injury; FA: fluid accumulation; APACHE II: Acute Physiology and Chronic Health Evaluation II score. Bold values represent a p-value < 0.05.

## Supplemental Figure 4: Full adjusted autoregressive linear mixed models showing the effect of different variables on serum creatinine values during the first 30 days of hospitalization for the different (A) CKD and (B) AKI stage subgroups


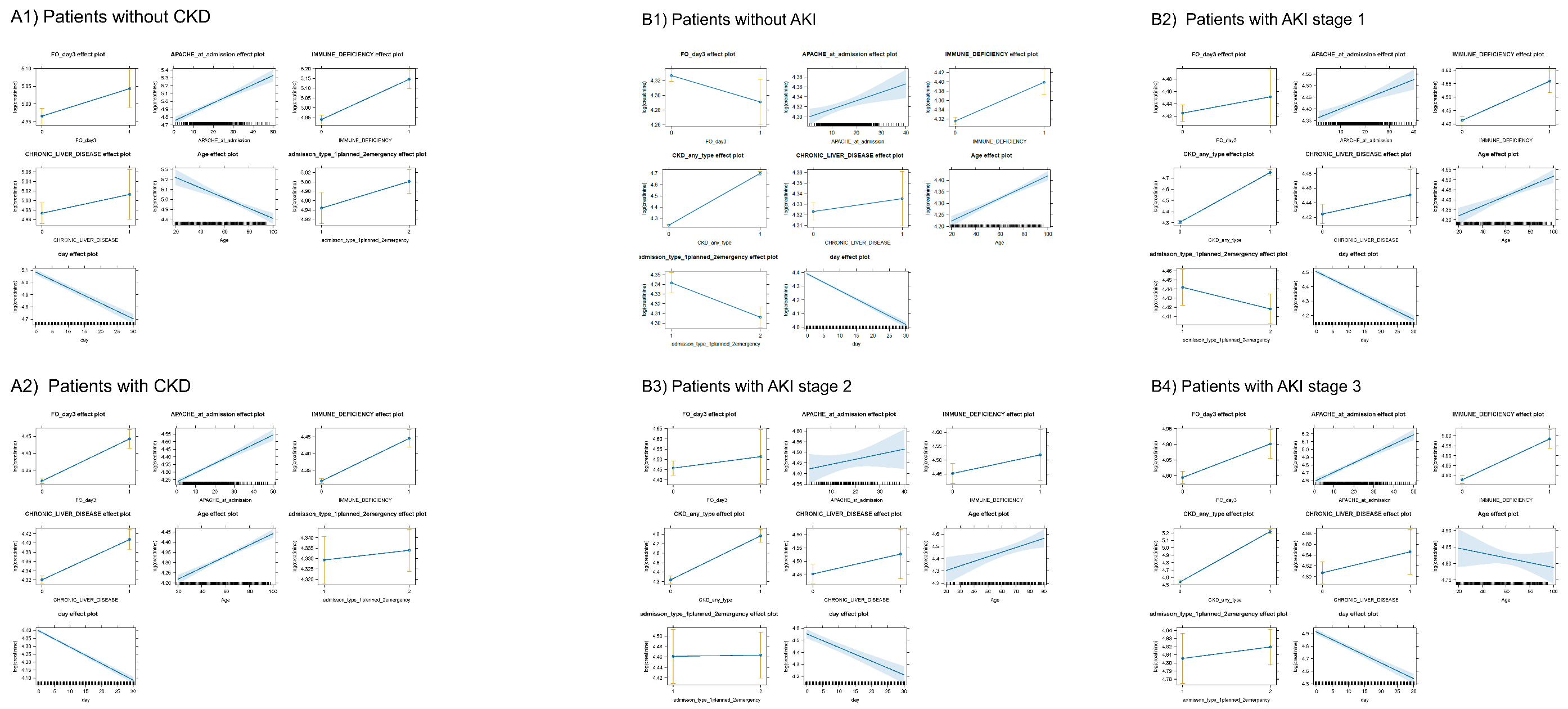

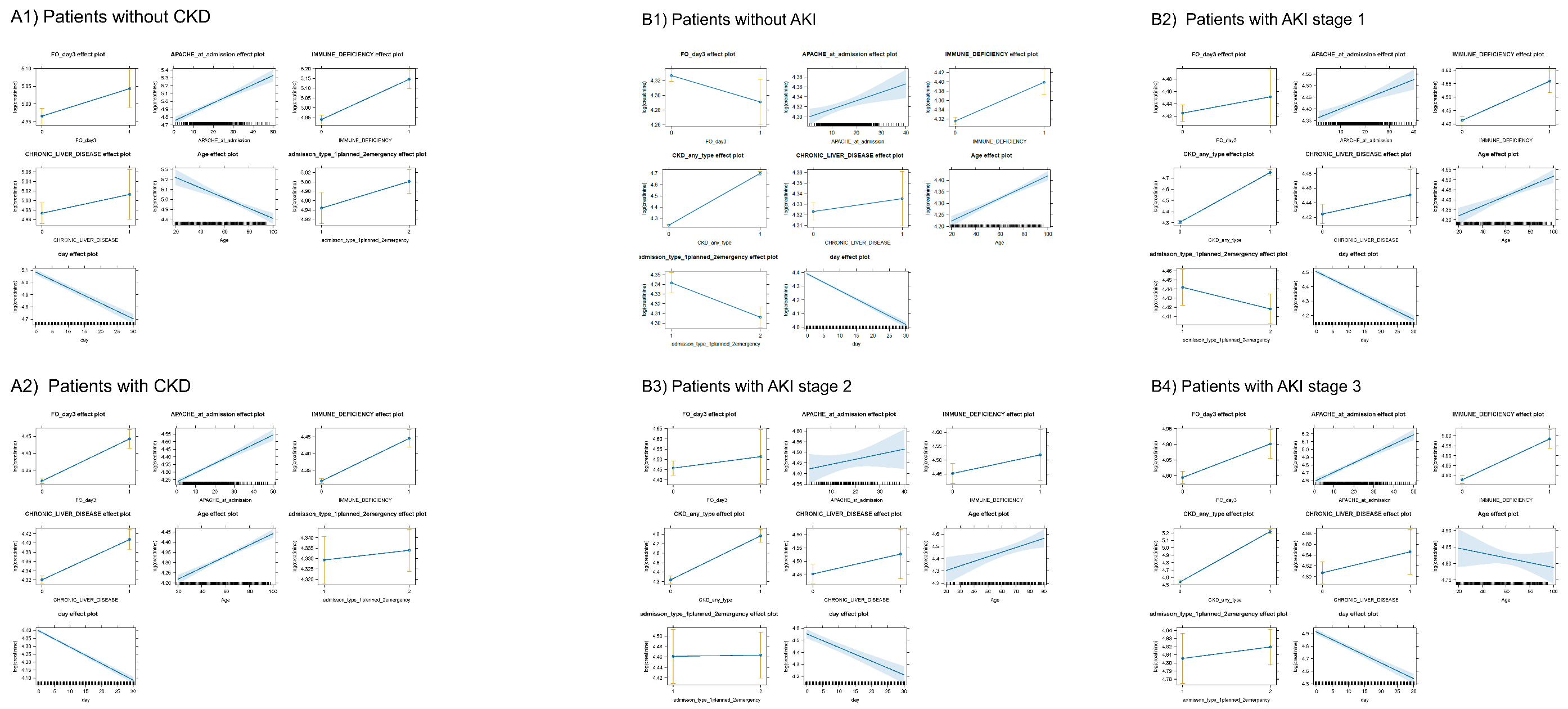


The black bars density along the horizontal axis represents the frequencies of the values in the data, the dark blue line the relative change between the intercept and the alternative categories across effects, while the light blue area (for numeric fixed effects) and the yellow bars (for categorical fixed effects) indicate the 95% confidence intervals of the model estimates.
CKD: chronic kidney disease; AKI: acute kidney injury; FA: fluid accumulation; APACHE II: Acute Physiology and Chronic Health Evaluation II score; CKD: chronic kidney disea
